# Biodegradation of 2,4-dinitrophenol with laccase immobilized on nano-porous silica beads

**DOI:** 10.1186/1735-2746-10-25

**Published:** 2013-04-01

**Authors:** Emad Dehghanifard, Ahmad Jonidi Jafari, Roshanak Rezaei Kalantary, Amir Hosein Mahvi, Mohammad Ali Faramarzi, Ali Esrafili

**Affiliations:** 1 Department of Environmental Health Engineering, School of Public Health, Tehran University of Medical Sciences, Tehran, Iran; 2 Department of Environmental Health, Faculty of Medical Sciences, Tarbiat Modares University, Tehran, Iran; 3 Center for Solid Waste Research (CSWR), Institute for Environmental Research (IER), Tehran University of Medical Sciences, Tehran, Iran; 4 Center for Water Quality Research (CWQR), Institute for Environmental Research (IER), Tehran University of Medical Sciences, Tehran, Iran; 5 Department of Pharmaceutical Biotechnology, Faculty of Pharmacy, Tehran University of Medical Sciences, Tehran, Iran

**Keywords:** Degradation, Laccase, Immobilization, Nano-porous silica beads, 2,4-dinitrophenol

## Abstract

Many organic hazardous pollutants, including 2,4-dinitrophenol (2,4-DNP), which are water soluble, toxic, and not easily biodegradable make concerns for environmental pollution worldwide. In the present study, degradation of nitrophenols-contained effluents by using laccase immobilized on the nano-porous silica beads was evaluated. 2,4-DNP was selected as the main constituent of industrial effluents containing nitrophenols. The performance of the system was characterized as a function of pH, contact time, temperature, pollutant, and mediator concentrations. The laccase-silica beads were employed in a mixed-batch reactor to determine the degradation efficiency after 12 h of enzyme treatment. The obtained data showed that the immobilized laccase degraded more than 90% of 2,4-DNP within 12 h treatment. The immobilization process improved the activity and sustainability of laccase for degradation of the pollutant. Temperatures more than 50°C reduced the enzyme activity to about 60%. However, pH and the mediator concentration could not affect the enzyme activity. The degradation kinetic was in accordance with a Michaelis–Menten equation with V_max_ and K_m_ obtained as 0.25–0.38 μmoles/min and 0.13–0.017 mM, respectively. The stability of the immobilized enzyme was maintained for more than 85% of its initial activity after 30 days. Based on the results, it can be concluded that high resistibility and reusability of immobilized laccase on CPC-silica beads make it considerable choice for wastewater treatment.

## Introduction

Nitrophenols, categorized as priority pollutants, are one of the main common components which release from industrial effluents and deteriorate the quality of water resources. There are six possible dinitrophenol (DNP) forms and 2,4-Dinitrophenol (2,4-DNP) is the most important toxic and refractory pollutant [1]. 2,4-DNP, a yellowish crystalline solid, has been used in manufacturing of pesticides, pharmaceuticals, production of dyes, explosive materials, and as an indicator for the detection of potassium and ammonium ions. Its entrance into the environment may occur from industrial wastewaters, accidental spills, or as an intermediate metabolite due to degradation of pesticides containing 2,4-DNP [2]. For instance, wastewater from a dye manufacturing plant contained 3.2 mg/L DNP. Groundwater from a waste site that was once occupied by a factory that used DNP contained 30.6 mg DNP/L of water [3].

Several physical and chemical methods which have been used for the treatment of the nitrophenols pollutants are adsorption processes, chemical oxidation, precipitation, evaporation, and incineration. However, due to their problems in the application and economic issues, other relevant treatment methods have been studied. The ability of biological processes on the degradation of organic pollutants, due to their effective and safe performance in compare with chemical and physical treatment techniques, has been considered [4,5]. Among them, the performance of white rots fungi for degradation a wide range of refractory organic pollutants, specially the phenolic compounds, via lignin-modifying enzymes, such as manganese (II)-dependent peroxidase, lignin peroxidase, and laccase (phenol oxidase) have been studied [6-8]. Although biological processes are efficient at low pollutant concentrations, their sensitivity to shock loads, require long hydraulic retention times and forming large amounts of solid residues make their feasible application with challenges [9]. In an enzyme based process, however, some of these disadvantages can be omitted since enzymes can be applied to persistent materials, high and low contaminant concentration over a wide pH, temperature, and salinity range. The most recent research in this area has focused on the enzymatic process for the treatment of wastewater [10,11].

Laccase (EC 1.10.3.2), a multi-copper oxidase enzyme, catalyzes the oxidation of variety of aromatic and inorganic substrates, mostly phenols, with simultaneous reduction of oxygen to water [12-14]. Among advantages of laccase application such as high efficiency, the main disadvantage is that it is often easily inactivated in oxidation process due to the wide range of process conditions (temperature, pH, etc.) and also its separation procedure, for reuse proposes, from the reaction system is difficult which limits the further industrial applications of laccase. An effective method for reuse and improving its stability is using enzyme immobilization technology [15]. Studies showed that several types of supporters could be used in enzyme immobilization which included activated carbon, chitosan microspheres [16], polymeric carrier [17], polyacrylonitrile beads [18] and magnetic chitosan nanoparticles [19].

Although many previous studies used immobilized laccase to degrade organic pollutants such as chlorophenols [20], dyes [21], Poly Aromatic Hydrocarbons (PAHs) [22], however there has not been a reliable research on oxidation of nitrophenol compounds by immobilized laccase. The aim of this study is to investigate the feasibility of 2,4-DNP degradation in effluents by *Trametes versicolor* laccase immobilized on the controlled porosity carrier (CPC) silica beads.

## Materials and methods

### Chemicals

*T. versicolor* laccase, pre-silanized [with 3-aminopropyltriethoxysilane (APTES)] CPC silica beads, Glutaraldehyde solution (25%), and 2,2^′^-azino-bis(3-ethylbenzthiazoline-6-sulfonic acid) (ABTS) were purchased from Sigma-Aldrich (St. Louis, MO, USA) and 2,4-DNP, acetonitrile and methanol (HPLC grade) were from Merck (Darmstadt, Germany).

### Laccase immobilization on CPC-silica beads

Laccase was immobilized on pre-silanized silica beads according to the study of Champagne and Ramsay [6]. An amount of 4 g of pre-silanized CPC-silica beads (355–600 mm in diameter, an average surface area of 42.1 m^2^/g, and a pore size of 37.5 nm) were immersed in degassed 2.5% glutaraldehyde (2.0 bar vacuum pressure for 2 h) in 0.1 M KH_2_PO_4_, pH 5.0, then placed in an enzyme solution (≈ 2.0 U/mL in 0.1 M KH_2_PO_4_ at pH 5.0) for more than 36 h at 4°C. Thereafter, beads were purred on a paper filter and washed three times with distilled water and twice with phosphate buffer (0.1 M KH_2_PO_4_, pH 5.0).

### Enzyme assay

Laccase activity was measured at 420 nm by generation of ABTS˚ˉ radicals from the enzymatic oxidation of ABTS at 25°C using CECIL 8600 spectrophotometer. The assay mixture contained 0.2 mM ABTS, 100 mM sodium acetate buffer (pH 5.0), and the enzyme-containing sample [18]. One unit of laccase activity (U) was defined as the amount of enzyme that formed 1 μmol ABTS per min. Protein concentration was measured as the absorbance at 280 nm and corrected for scattering effects with absorbance readings at 320 nm [6].

### Degradation of 2,4-DNP by immobilized laccase

For determining the performance of immobilized enzyme on 2,4-DNP degradation, 0.5 g of CPC-silica beads with 50 ± 3.8 U of laccase/g (i.e. 1.18 ± 0.09 U/m^2^) were used in a batch reactor (Erlenmeyer 50 mL). Synthetic effluent consisted of 2,4-DNP with concentrations of 0.05, 0.1, and 0.15 M (in 0.1 M phosphate buffer, pH 4–6) and ABTS (as a mediator) of 1, 2, and 3 mM was added to each reactor. Temperature had been adjusted in range of 40–60°C in the shaker incubator (150 rpm). The sampling procedure (1 mL for every 2 h up to 12 h retention time) was done, and then equal volume of methanol (HPLC grade) was added in order to end the reaction process between enzyme and the pollutant. Samples were then filtered by 0.2 μm PTFE (Polytetrafluoroethylene) filters and filled in 5 mL dark vessels (sealed by parafilm tape) in 4°C before analysis.

### Analysis and measurements

The concentration of 2,4-DNP in the reaction mixture was measured by high performance liquid chromatography (HPLC, CECIL 4100, USA) equipped with an UV–Visible Diode Array detector. Separation of compounds was obtained with a Nucleodur Sphinx RP (25.0 cm × 4.6 mm) column (MZ-1 PerfectSil, Germany) at a flow-rate of 1 ml/min. The chromatographic determination was performed by using a gradient in 10 min acetonitrile/ 0.5% acetic acid (50:50, v/v). All assays were carried out in triplicate and gave standard deviations lower than 5%.

## Results and discussion

### Characterization of CPC-silica beads

**Figure 1:**
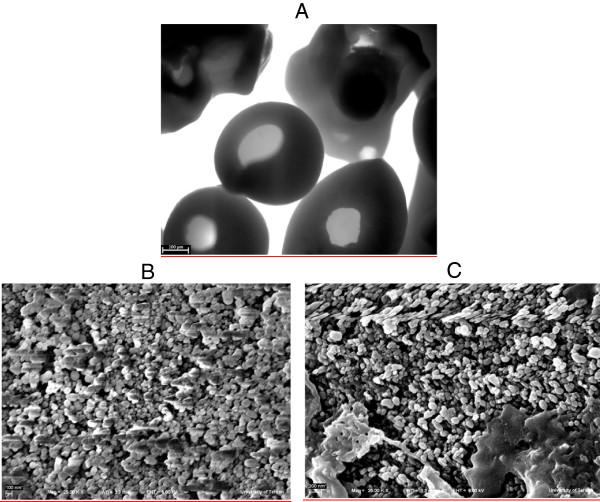
SEM imaging of nanoporous cpc-silica beads, A) overall shape of beads, B) the surface of beads without immobilized enzyme, C) the surface of beads with immobilized enzyme.

The morphology of CPC-silica beads which was acquired using SEM is shown in Figure 1. It must be noted that the beads were purchased and no additional activity was done for preparing that. As demonstrated in Figure 1A, most silica beads had regular convoluted structure at the micron scale. However, the surface of silica beads had many pores which most of them had diameters less than 100 nm. The nitrogen adsorption–desorption isotherm data indicate that the surface area of silica A is about 40 ± 5 m^2^/g.

As shown in Figure 1C, it could be observed that the laccase enzyme was effectively immobilized on the surface of the beads due to high surface area which the beads provided. In particular the nano-scale pores and the surface properties provided the suitable prerequisites for enzyme immobilization [6]. By comparison of Figure 1B and C, the immobilized laccase was obviously detectable.

### Characteristics of free and immobilized laccase

**Figure 2:**
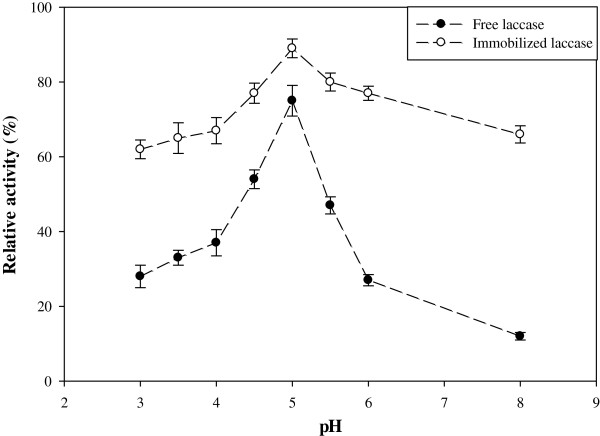
Relative activity of free and immobilized laccase in different pH [ABTS] = 1 mM; [temperature] = 40°C.

Activity dependence of immobilized laccase on temperature and pH values was determined and compared with the free enzyme. The oxidation reaction for immobilized laccase had the maximum activity at pH 5.0. As demonstrated in Figure 2, the pH trend of immobilized laccase was more extended in comparison with the free form, which showed that the immobilized laccase was more resisting to pH variations. In the pH range of 4–6, the immobilized laccase retained its activity for more than 86% of its maximum activity, however for the free form it was 36%. Moreover, pre-test analysis in pH 3.0 showed that the activity of immobilized enzyme versus free type retained 62% and 28%, respectively, and in alkaline pH (pH ≈ 8.0) the immobilized laccase kept its activity about 60–70% of its maximum activity, while free laccase was almost inactive. Catapane et al. also showed that the optimal pH for free and immobilized laccase on PAN (Poly Acrylo Nitrile) was about 5, however the activity of immobilized enzyme in pH range of 3 to 7 was retained significantly, in compare with free enzyme [23].

**Figure 3:**
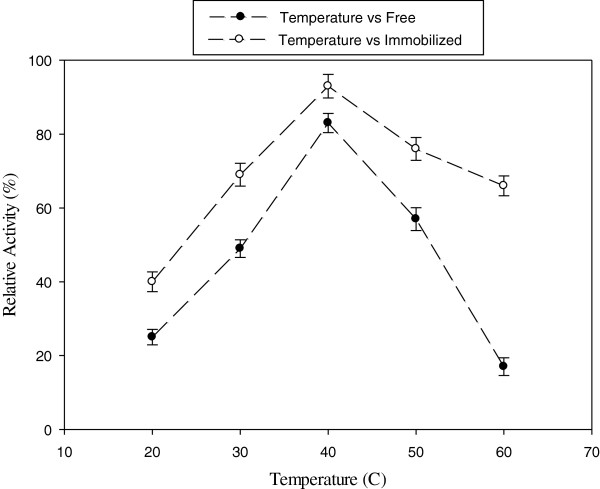
The effect of temperature on relative activity of free and immobilized laccase [ABTS] = 1 mM; [pH] = 5.

For determining the effect of temperature on laccase, the activity of free and immobilized enzyme was assayed in a solution containing 1 mM of ABTS, a laccase substrate, in 0.1 M phosphate buffer at pH 5.0 in the temperature range 20–60°C. As it can be seen in Figure 3, the maximum activity of both free and immobilized laccase was at 40°C. Moreover, the activity of the immobilized laccase was greater in high temperatures (50–60°C) compared with the free counterpart. Several studies have shown the temperature resistance of immobilized enzyme in contrast with free form [6,18,24]. Results demonstrate that the activity of immobilized enzyme in 60°C retains about 70% of its maximal activity, while the free enzyme is inactivated. Nicolucci et al. also showed that the relative activity of immobilized laccase was about 80% at 70°C while the free laccase was approximately inactivated [18].

### Enzymatic degradation of 2,4-DNP

**Figure 4:**
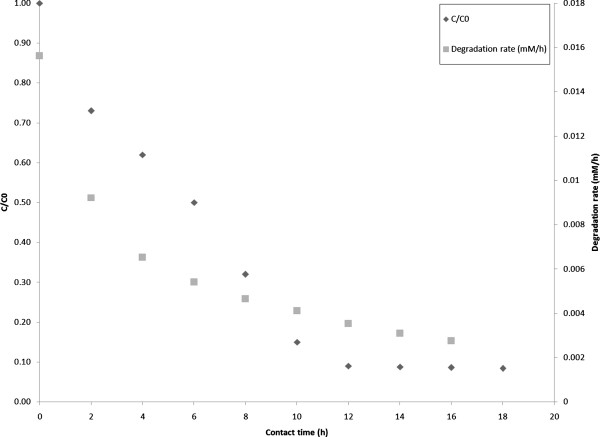
Residual concentration and degradation rate of 2,4-DNP in different contact time [2,4-DNP] = 0.05 mM; [ABTS] = 1 mM; [temperature] = 40°C; [pH] = 5.

Enzymatic degradation studies of 2,4-DNP were conducted in a batch reactor which CPC-silica beads were easily spread in a reactor by shaking by the rate of 150 rpm. Results showed that the immobilized laccase could effectively break down 2,4-DNP (Figure 4). The maximum degradation of 2,4-DNP was raised to 91% on 12 h contact time at 40°C (pH 5, ABTS 3 mM) which was selected as the optimum condition. It must be noted that rising the contact time over 12 h could not significantly increase the degradation efficiency (P_value_ < 0.05). It is obvious that the contact time plays an important role in pollutant degradation which increases the reaction. Bhattacharya and Banerjee confirmed the positive effect of contact time of enzymatic degradation of 2,4-DCP [25]. However, the degradation rate of the pollutant had decreasing trend from 0.015 to 0.002 mM/h which may due to enzyme inactivation or synthesizing of intermediate compounds during oxidation of 2,4-DNP and therefore laccase involvement to degrade those chemicals [18].

**Figure 5:**
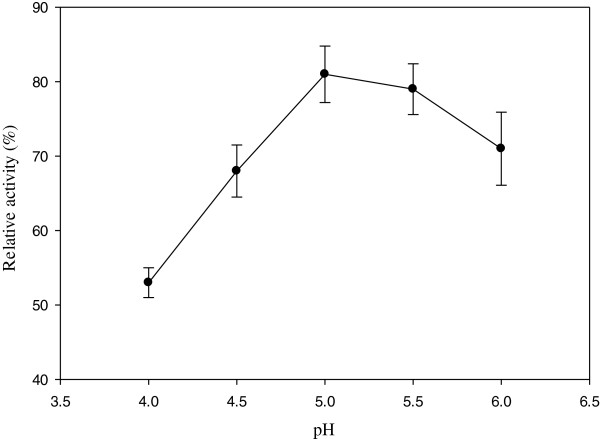
Relative activity of the immobilized laccase in different pH for 2,4-DNP [2,4-DNP] = 0.05 mM; [ABTS] = 1 mM; [temperature] = 40°C.

As demonstrated in Figure 5, a bell-shaped dependence on pH was appeared for the plot of 2,4-DNP degradation which is in accordance with the pH profile of laccase activity. This result is consistent with earlier literature reports [26,27]. The optimum pH of 5 was achieved for enzymatic degradation of the pollutant that is in agreement with other researches [26,28]. However, the optimum pH reported by Okazaki et al. was 3 for laccase from *Carialus versicalar* converting α-phenylenediamine. This difference may be due to differences in the type and concentration of the buffer used and the purity of the enzyme [27,29]. Nicolucci et al., revealed that laccase activity was higher in acidify medium [18]. Moreover, immobilized laccase on silica beads retained its activity in pH 4–6.2 [29].

**Figure 6:**
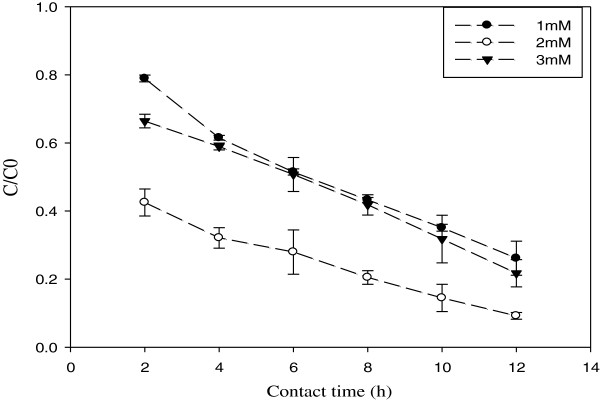
The effect of mediator concentration on the degradation of 2,4-DNP [2,4-DNP] = 0.05 mM; [temperature] = 40°C; [pH] = 5.

Laccase normally has low redox potential (0.6–0.8 V) which is limited to react with wide range of phenolic compounds [30-32]. However, using proper low molecular weight compounds, known as mediators, can mediate the oxidation of substrates by laccase, so extending the application of laccase on oxidation of wide range of pollutants [6,18]. As shown in Figure 6, the acceptable role of a mediator is to act as an “electron shuttle” between laccase and the substrate [33]. The oxidized mediator by laccase, can strongly oxidizes the substrate due to its higher redox potential (1.8 V for ABTS) [34]. The results in Figure 6 showed that increasing the mediator concentration from 1 to 3 mM could enhance the conversion of 2,4-DNP, however, its effect was calculated as 2%. During the oxidation of 2,4- DNP, the color of the system gradually changed from blue-green to light yellow which indicated ABTS^++^ was probably present in the systems of this study. Liu showed that increasing ABTS concentration from 10 to 200 μM enhance the conversion of Bisphenol A [29].

Temperature is an important variable to be considered in laccase-catalyzed reactions and has a double effect on enzymatic systems which are a change in the rate of reaction over time caused by thermal inactivation of the enzyme, and a change in the reaction rate due to Arrhenius effects [8]. Results revealed that by increasing temperature from 40 to 60°C, the pollutant degradation significantly decreased from 91% to only 33%, while other parameters kept constant (optimum condition). In compared with the resistance of free enzyme on temperature, as previously noted, its remained activity could be explained with the immobilization process and improving laccase characteristics [8]. Tavares et al. showed that the activity immobilized laccase on modified silica beads was remained for more than 6 h in 55°C and by increasing the temperature up to 70°C, the enzyme was almost deactivated [35].

**Figure 7:**
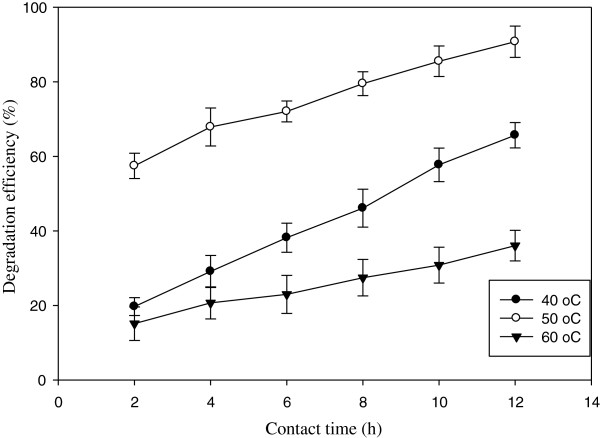
The degradation of 2,4-DNP in different temperatures [2,4-DNP] = 0.05 mM; [ABTS] = 1 mM; [pH] = 5.

As can be seen in Figure 7, the activity of laccase for 2,4-DNP degradation was remained in higher temperature (60°C) in accordance with different studies which have reported the stability of laccase in wide range of high temperatures even in 100°C [29].

### Kinetics of 2,4-DNP degradation

The initial reaction rate of 2,4-DNP was measured for determining the kinetic parameters of enzyme reactions. A Michaelis-Menten equation was used for data fitting process. Lineweaver–Burk double reciprocal plots were applied to calculate K_m_ and maximum reaction rate (V_max_) of the immobilized laccase. As shown in Table 1, the K_m_ values of the immobilized laccase for different concentrations of 2,4-DNP (0.05, 0.1, and 0.15 mM) resulted to 0.013, 0.016, and 0.017 mM, respectively. A general increase in the K_m_ values for the immobilized laccase towards every substrate concentration was observed. On the contrary, a decrease in V_max_ values was observed. The V_max_ for immobilized laccase was reduced from 0.38 to 0.25 μmoles/min by substrate increasing. Due to variation of both K_m_ and V_max_ values with substrate concentration, the values of V_max_/K_m_ are also reported in Table 1 for easy comparison of the catalytic efficiency of enzyme-substrate system. The ratio varied between 29.23 and 14.70 for the immobilized laccase. The decreasing trend of the ratio V_max_/K_m_ may be occurred due to inactivation of the enzyme and the production of intermediate compounds during degradation of 2,4-DNP [18].

**Table 1:** Kinetic parameters for the immobilized laccase using 2,4-DNP as substrates

**Substrate concentration (mM)**	**V **_**max **_**(μmoles/min)**	**K**_**m **_**(mM)**	**V **_**max/Km **_**(μmoles/min/mM)**
0.05	0. 38	0.013	29.23
0.10	0. 31	0.016	19.37
0.15	0. 25	0.017	14.70

### Stability of immobilized enzyme

**Figure 8:**
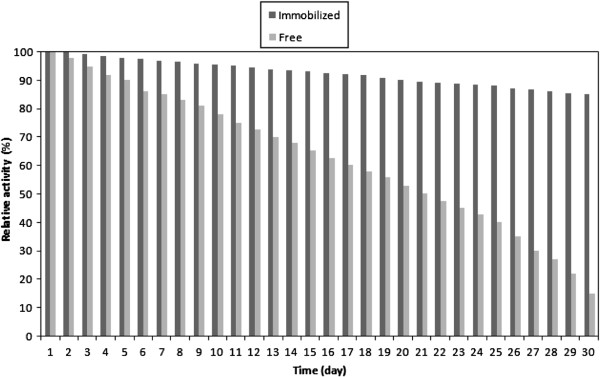
Comparison of the stability of free and immobilized enzyme [ABTS] = 1 mM; [temperature] = 40°C; [pH] = 5.

Operational stability is considered as an important factor in order to evaluate the industrial application of enzymes. The immobilized laccase on silica beads were reused every day for 30 successive days. Duration of each experiment was 12 h. The activity of the immobilized enzyme was assayed daily by a spectrophotometrically measurement of color change of the mediator. The experiment condition was the same in the period as 1 mM of ABTS in phosphate buffer solution 0.1 M, pH 5.0 at 25°C. As depicted in Figure 8, the immobilized laccase retained its activity for more than 85%. However, the activity of free laccase remained only 15% of its initial activity. The results confirmed that the immobilized laccase on nano-porous silica could effectively retain its activity and stability [6,23,35].

## Conclusions

In this study, the performance of the immobilized laccase for degradation of 2,4-DNP in aqueous solutions has been investigated. Results showed that the immobilized enzyme was able to effectively degrade 2,4-DNP as a pollutant in water resources. The optimum condition for the maximum degradation of 2,4-DNP (91%) was achieved on 12 h contact time and pH 5. The immobilized laccase had more resistibility to the pH and temperature changes, in compare with the free form. The improved characteristics of immobilized laccase on CPC-silica beads for stability and reusability could be considered as an advantage in wastewater treatment. Investigation of the enzymatic degradation of other phenolic compounds with several mediators in different pH and Temperature condition could be recommended.

## Competing interests

The authors declare that they have no competing interests.

## Authors’ contributions

The overall implementation of this study including design, experiments and data analysis, and manuscript preparation were the results of efforts by corresponding author. All authors have made extensive contribution into the review and finalization of this manuscript. All authors read and approved the final manuscript.
